# Recent Advances in ZnO Nanomaterial-Mediated Biological Applications and Action Mechanisms

**DOI:** 10.3390/nano13091500

**Published:** 2023-04-27

**Authors:** Jiani Xie, Huilun Li, Tairan Zhang, Bokai Song, Xinhui Wang, Zhanjun Gu

**Affiliations:** 1 College of Food and Biological Engineering, Chengdu University, Chengdu 610106, China; 2 CAS Key Laboratory for Biomedical Effects of Nanomaterials and Nanosafety, Institute of High Energy Physics, Chinese Academy of Sciences, Beijing 100049, China; 3 Clinical Medical College, Chengdu University, Chengdu 610106, China

**Keywords:** ZnO, nanomaterials, biological applications, action mechanism

## Abstract

In recent years, with the deepening research, metal zinc oxide (ZnO) nanomaterials have become a popular research object in the biological field, particularly in biomedicine and food safety, which is attributed to their unique physicochemical properties such as high surface area and volume ratio, luminescence effect, surface characteristics and biological activities. Herein, this review provides a detailed overview of the ZnO nanomaterial-mediated biological applications that involve anti-bacterial, anti-tumor, anti-inflammation, skin care, biological imaging and food packaging applications. Importantly, the corresponding action mechanisms of ZnO nanomaterials are pointed. Additionally, the structure and structure-dependent physicochemical properties, the common synthesis methods and the biosafety of ZnO nanoparticles are revealed in brief. Finally, the significance and future challenges of ZnO nanomaterial applications are concluded.

## 1. Introduction

**Figure 1:**
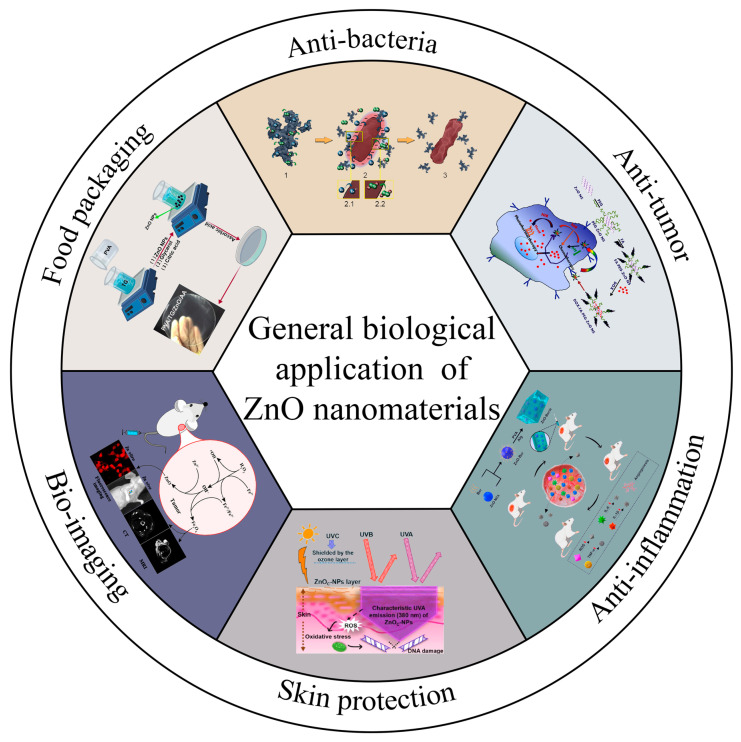
The biological applications of ZnO nanomaterials. Anti-bacteria: Adapted with permission [7]. Copyright 2019, Elsevier B.V. Anti-tumor: Adapted with permission [8]. Copyright 2017, Elsevier B.V. Anti-inflammation: Adapted with permission [9]. Copyright 2022, Frontiers Media S.A. Skin protection: Adapted with permission [10]. Copyright 2022, Elsevier B.V. Bio-imaging: Adapted with permission [11]. Copyright 2017, Springer Nature Switzerland AG. Food packaging: Adapted with permission [12]. Copyright 2020, Elsevier B.V.

Nanoparticles are a kind of tiny particle with a particle size between 1 and 100 nm, including zero-dimensional structures (quantum dots and nanoclusters), one-dimensional structures (nanotubes and nanorods), two-dimensional structures (thin films) and three-dimensional structures (composites and nanofibers, etc.) [1]. The size of semiconductor inorganic particles down to the nanoscale significantly affects the physical, chemical and biological activity of the particles. ZnO is a multifunctional semiconductor material with a wide bandgap (3.37 eV) and large excitation binding energy (60 mV) at room temperature [2]. As more and more functions of ZnO nanomaterials are discovered, their main applications have expanded from industrial manufacturing to the biological field. In the background of antibiotic abuse and increased bacterial resistance nowadays, the multifunctional characteristics of ZnO nanoparticles, such as high levels of reactive oxygen species (ROS) production, inhibition of inflammatory factor release and inhibition of mast cell degranulation, make them a new promising candidate for anti-bacterial and anti-inflammatory drug development [3,4,5,6]. Meanwhile, their good antibacterial efficacy means they can be effectively extended to food packaging applications, and they are a well-documented Food and Drug Administration (FDA)-approved material [1]. Next, the large specific surface area, high drug loading rate, high selectivity and low toxicity of ZnO nanoparticles make them ideal drug delivery systems for targeted therapy, particularly for cancer therapy. Additionally, ZnO nanoparticles can absorb a large amount of UV light and thus have been widely used in sunscreen products. Furthermore, owing to their photoluminescence performance, ZnO nanoparticles have been widely used in bioimaging, which provides new tools for biomedical diagnosis. Thus, it seems that ZnO nanoparticles are already everywhere in daily life. Nowadays, studies on the biological applications of these ZnO nanomaterials are common, but a more comprehensive overview of them with a systematic mechanism summarization is still rare. Therefore, this review provides an overview of ZnO-based nanomaterials from action mechanisms to applications in biological fields. Firstly, the structure, as well as structure-dependent physicochemical properties and the common synthesis methods of ZnO nanoparticles, are revealed in brief. Then, the biological applications and action mechanisms of ZnO nanoparticles are summarized in detail, which involve anti-bacterial, anti-tumor, anti-inflammatory, bioimaging, skin care and food packaging applications (Figure 1). In addition, the biosafety of ZnO nanoparticles is mentioned. The hope is that such a review can help researchers to fully understand the progress and status of the field so as to promote future developments.

## 2. The Structure and Structure-Dependent Properties of ZnO Nanoparticles

**Figure 2:**
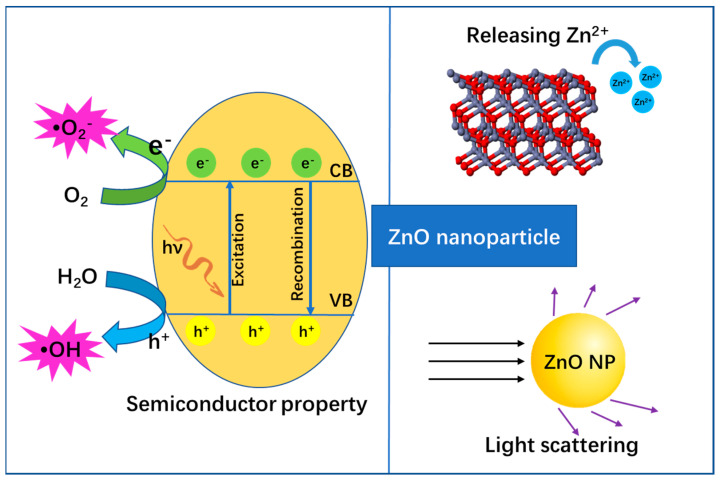
The structure-dependent properties of ZnO nanoparticles.

It is well-known that ZnO is a typical semiconductor with a wurtzite crystal structure, which is the basis for its unique properties such as catalytic, electrical and optical properties [13]. For example, as seen in Figure 2, when ZnO is excited by light (e.g., ultra-violet (UV)), electron(e^−^)-hole(h^+^) pairs are generated, where the excited e^−^ will jump into the conduction band (CB) and h^+^ is left in the valence band (VB). Subsequently, free radicals continuously generate from both the reductive pathway (e^−^) and oxidative pathway (h^+^), where the e^−^ transfers to an acceptor (often O_2_) to produce •O_2_^−^ and h^+^ transfers to a donor (often H_2_O) to produce •OH [14]. Both •O_2_^−^ and •OH are physiologically relevant toxic ROS, which can attack biomacromolecules (e.g., DNA) and cell components [15]. Herein, this property endows ZnO nanoparticles with the ability for anti-bacterial and anti-cancer biomedical applications, etc. On the contrary, recombination of the e^-^–h^+^ pair emits photons, which allows ZnO nanoparticles to be applied in bio-imaging [14]. Next, as a metal oxide, ZnO can slowly release the metal ion of Zn^2+^ in a complex external environment. Such a metal ion can interact with protein, and thus can possibly inhibit enzyme activity to cause physiological dysfunction and eventually lead to cell death. Meanwhile, structural characteristics such as suitable size, surface charge effect, abundant surface modification and high specific surface area endow ZnO nanoparticles with multiple functions such as ready cell ingestion, target identification and efficient drug delivery. Therefore, these properties are also beneficial to a variety of biotherapy applications (e.g., anti-bacterial, anti-cancer, anti-inflammatory). In addition, the nano-sized ZnO possesses a light scattering and reflecting function, which is closely related to its sunscreen applications. In a word, the unique structural characteristics of ZnO nanoparticles determine its multifunctional performance and ultimately give its potential for rich biological applications.

## 3. The Common Synthesis Methods of ZnO Nanoparticles

Considering the large demand for ZnO nanoparticles for industry and living applications, the development of new synthesis methods to prepare uniform nano-sized ZnO is of great significance for both fundamental study and practical application. To date, a wide variety of ZnO nanoparticle preparation methods have been put forward, from physicochemical synthesis to green biogenic synthesis. Initially, physicochemical synthesis was the common preparation means, which mainly includes the methods of precipitation [16,17,18,19,20,21], electrochemical deposition [22,23,24], hydrothermal synthesis [25,26,27,28,29,30,31,32], sol-gel process [33,34], solid state reaction [35,36], chemical vapor synthesis [37], gel combustion [38], microemulsion [39], solvothermal synthesis [40,41,42], spray pyrolysis [43], sputtering [44,45,46], sonication [47,48], microwave [49], thermal evaporation [50,51,52] and so on. Each of these methods has its own advantages. Typically, the precipitation method is synthetically controlled. Additionally, it does not require complicated equipment and expensive raw materials, and thus is low cost and suitable for large-scale production [16,47]. Spray pyrolysis is suitable for large area preparation in a short span of time and its instrumentation is simple [43]. The solvothermal method is low-cost, simple, catalyst-free and seed-free [42]. The microwave approach has a high reaction selectivity, short reaction time, rapid volume heating and is energy-efficient [49]. The sol-gel way has the superiority of simplicity, repeatability and reliability. Importantly, the products of the sol-gel method exhibit the characteristics of high purity, low particle size and high specific surface area [21,53]. Hydrothermal synthesis is simple and can operate in a controlled manner, which is helpful to tailor the particles’ morphology via controlling the reaction rates of hydrolysis and condensation [53]. Moreover, the electrochemical method also is cost-effective and has a high purity of the final products. In particular, it is a low temperature operation process [22,23]. It is worth mentioning that in addition to their advantages, disadvantages also exist among these methods. For instance, the common physical methods of sputtering and thermal evaporation are expensive and consume high amounts of energy [19]. Traditional chemogenic syntheses such as hydrothermal synthesis, sol-gel process, chemical vapor synthesis, gel combustion and solvothermal synthesis usually need a high temperature atmosphere, and some of them must even be carried out in an expensive inertia environment. More seriously, most of them need toxic reagents or additives in the reaction process. As a result, the high temperature, complexity and toxicity of these methods limit their widespread application [17,36]. In this respect, our member once employed a catalyst-free and low-temperature oxidization method to prepared ZnO nanorod arrays [54]. The ZnO nanorod arrays were controlled growth on metal zinc foils and did not affect the conductive function of the zinc foils. The control of oxygen introduction played a key role in the success of this synthesis. This work provides great opportunities to investigate the effects of the material structure of these properties. Recently, an emerging concept named green synthesis has been highly preferred owing to its advantages of biocompatibility, biosafety, eco-friendliness, cost effectiveness and easiness to large-scale synthesis as compared with physicochemical methods [55,56]. The green synthesis of ZnO nanoparticles uses biological components as raw materials, such as plants extract, microorganisms and other bio-regenerable materials, which are regarded as excellent renewable sources. Additionally, the key mechanism of them is that the biomolecules derived from biological components are active compounds that serve as reducing and capping agents [55,57]. Currently, with cutting-edge developments in science and technology such as artificial intelligence, we believe that the potential of green synthesis can be more fully developed, and more and more new synthesis methods will emerge in the near future.

## 4. Bio-Application and Action Mechanisms of ZnO Nanomaterials

### 4.1. ZnO Nanomaterials for Anti-Bacterial and Anti-Fungal Applications

Both bacteria and fungi are microorganisms. The difference is, bacteria have no nucleus surrounded by nuclear membrane, which means they belong to prokaryotes, and fungi do have this, and belong to eukaryotes. The development of efficient bactericide and fungicide is of great significance in many fields such as oral care and cleansing, medical bandages, anti-infection, wound healing and plant pathogen-induced crop loss treatment. Herein, this section will introduce in detail the potential of ZnO nanoparticles for anti-bacterial and anti-fungal applications. It is worth mentioning that the anti-bacterial and anti-fungal mechanisms of ZnO nanoparticles are similar; we thus just introduce the anti-bacterial mechanism here.

#### 4.1.1. Anti-Bacterial Mechanisms

**Figure 3:**
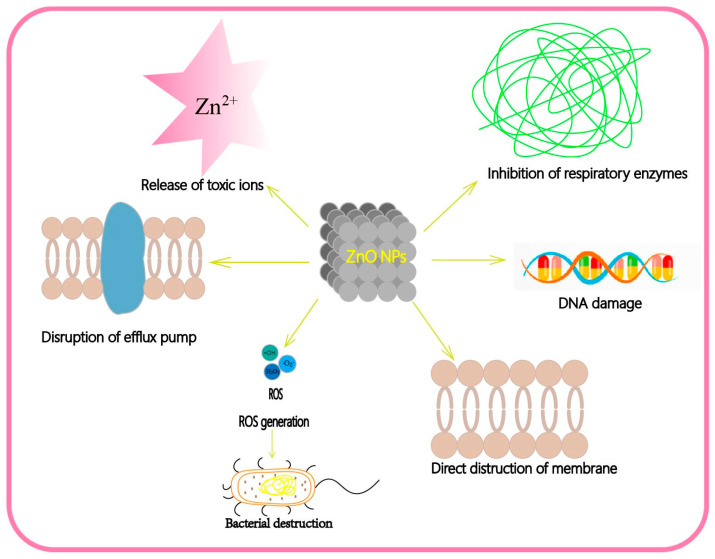
The anti-bacterial mechanism of ZnO nanomaterials.

ZnO is currently considered one of the most promising inorganic antimicrobial agents due to its low cytotoxicity, high selectivity and good heat resistance [3]. Although it has certain antibacterial activities, its antibacterial mechanism is not fully understood. To date, the existing view is that ZnO can cause bacterial death through direct damage to cells or indirect generation of ROS to inhibit protein function, destruct nucleic acid and produce harmful lipid peroxidation in bacteria [58]. By searching the literature, we summarize the main antibacterial mechanisms of ZnO nanoparticles as follows (Figure 3): (1) The direct interaction of nanoparticles with bacterial membrane leads to the destruction and rupture of the membrane’s integrity [59]. (2) Generating ROS in the cell to destroy the bacteria [4]. (3) Release of toxic Zn^2+^ ions to kill bacteria. Zn^2+^ has a special affinity to bacterial sulfhydryl groups. It can bind and oxidize the groups to inhibit glycolytic enzyme activity, and thereby inhibit the glycolytic pathway [60]. Thus, it can inhibit the action of bacterial respiratory enzymes and eventually lead to cell death. What is noteworthy is that for different types of bacteria, ZnO nanomaterials enter the bacteria in different ways. For Gram-positive bacteria, the Zn^2+^ in ZnO nanomaterials can be chelated by teichoic acid and lipoteichoic acid of the cell and translocate into cell. For Gram-negative bacteria, it has the porins that act as ion channels to promote passive diffusion into cell. (4) Disruption of the efflux pump and membrane potential on the cell membrane [61]. (5) Internalization of ZnO leads to intracellular DNA damage.

#### 4.1.2. Anti-Bacterial and Anti-Fungal Applications

The size, morphology and even synthetic method of the nanoparticles will affect their antibacterial properties. Typically, the smaller particle size endows ZnO nanoparticles with the ability to more easily penetrate bacteria, and thus their antibacterial activity is stronger [3]. Based on this characteristic, many researchers have controlled the size of ZnO nanoparticles using various methods to improve their antibacterial activity. Lallo da Silva et al. obtained ZnO nanoparticles using the sol-gel method and used (3-glycidyloxypropyl) trimethoxysilane (GPTMS) as surface modifier [3]. The experimental results showed that the particle size was smaller and the antimicrobial performance of the material was stronger. Additionally, ZnO nanoparticles with a size of about 5 nm exhibited excellent anti-microbial activity. Verma et al. produced ZnO nanocrystalline via high energy ball milling (HEBM) and investigated the effect of size-dependent core–shell intrinsic defects to antibacterial activity [62]. The experimental results showed that the core shrunk in the ZnO core–shell structure with a decrease in size. Meanwhile, the antibacterial activity had a negative correlation between bacterial viability and the size of ZnO. Additionally, the morphology of the nanoparticles also affected their antibacterial activity. For example, Sharma et al. synthesized ZnO nanoparticles with various morphologies using an eco-friendly bio-mediated solution combustion method using different doses of *Carica papaya* milk (CPM) latex as fuel[59]. Interestingly, ZnO nanoparticles with the morphology of nano-flowers exhibit excellent photocatalytic activity over the other ZnO nanoparticles. As a result, ZnO nano-flowers manifest powerfully antibacterial activity against *Staphylococcus aureus* and *Pseudomonas aeruginosa*. Similarly, like the eco-friendly bio-mediated synthetic method above, researchers found that the synthesis of ZnO nanoparticles associated with plant extracts can obtain materials with ideal antibacterial effects, where plant-mediated ZnO green syntheses such as *Psidium guajava* leaf extract [63], *Pedalium murex* plant extract [64], *Artemisia haussknechti* leaf extract [65], *Parthenium hysterophorus* leaf extract [66], *Gymnema sylvestre* leaf extract [67], *Aloe vera* and *Hibiscus sabdariffa* plant extracts [68] have been reported. In addition, the synthetic method of ZnO nanoparticles is also closely related with their antibacterial activity. A typical example is that Mahendiran et al. proved that among all the ZnO nanoparticles they prepared, the best bactericidal activity was obtained with ZnO nanoparticles synthesized with biological methods rather than chemical methods [68].

Recently, researchers found that the applications of ZnO nanoparticles are limited due to their wide energy band gap and the high complexation of photogenerated electron-hole pairs. Fortunately, metals’ hybridization to form a complex, such as a combination of silver, gold, or palladium with metal oxides, can reduce their band gap [69]. Nguyen et al. synthesized silver-decorated ZnO nanomaterials, which made the absorption edge shift to the lower energy region of oxide nanoparticles and improved the antibacterial performance of the materials against *Staphylococcus aureus* (Gram-positive) and *Escherichia coli* (Gram-negative) bacteria [69]. In addition to the doping of other metal ions, ZnO nanomaterials are also widely used when compounded with other non-metal inorganic materials, such as ZnO/graphene oxide composites [2]. Compared with individual ZnO nanomaterial or graphene oxide alone as an antibacterial agent, the electrons in the ZnO/graphene oxide composite can be rapidly transferred between ZnO particles and graphene oxide, which facilitates the formation of ROS to disrupt the bacterial cell membrane, and thus enhances the antibacterial properties. Furthermore, ZnO nanomaterials can also be compounded with organic materials, such as Chitosan [70]. Chitosan is more widely used as a stabilizer due to its good biocompatibility and low toxicity. More importantly, the chitosan amine group possesses a positive charge, which promotes the complexation of chitosan-stabilized ZnO nanoparticles with the cell surface (negative charge), and thus produces a strengthened antibacterial effect.

In addition to anti-bacterial applications, research also shows that ZnO nanoparticles can be used as a fungicide [71,72,73,74,75,76,77,78,79,80,81,82]. For example, Khan et al. reported thorn-like ZnO nanoparticles via sol-gel synthesis, which showed an effective antifungal effect to *Candida albicans* [75]. Sharma et al. prepared ZnO nanoparticles by a simple homogeneous precipitation method without adding any chelating, surfactant or gelating agents. It was found that the antifungal activity against *Candida albicans* was enhanced with the increased concentration of ZnO nanoparticles [80]. Krishnamoorthy et al. prepared ZnO nanoparticles using *Citrus hystrix* leaf extract. The nanoparticles exhibited good antifungal activity against *Candida albicans* and *Aspergillus niger* at their higher concentration of 5 μg/mL [76]. Moreover, Ahmed et al. prepared ZnO nanoparticles using *Azadirachta indica* leaf extract for biocontrol of diseased lychee fruits. The disease-causing pathogens were identified as *Aspergillus niger*. In vitro results indicated that maximum mycelial growth inhibition was 70.5% under the ZnO nanoparticles (1.0 mg/mL). In vivo results obtained maximum suppression of lychee fruit rot under the same concentration [72]. Recently, in order to further improve the antifungal activity, ZnO nanocomposites have gradually come into researchers’ perspectives instead of pure ZnO nanoparticles. As an example, Roy et al. used ZnO nanoparticles, montmorillonite (MMT) and high density polyethylene (HDPE) to prepare HDPE/ZnO-MMT nanocomposites. This nanocomposite presented significant antifungal properties against black mold fungi *Aspergillus niger* at 5 wt% of loading [79]. Gondal et al. synthesized 5%Pd-doped nano-ZnO and compared its antifungal activity with that of pure nano-Zn. It demonstrated that the 5%Pd-doped nano-ZnO and pure nano-ZnO exhibited antifungal effects against *Aspergilus niger* with a minimal inhibitory concentration (MIC) of 1.25 and 2.5 mg/mL, respectively, *Candida albicans* yeasts with an MIC of 2.5 and 5 mg/mL. Therefore, the loading of nano-ZnO with Pd increased the antifungal activity compared with pure that of nano-ZnO [73]. Haghighi et al. evaluated the light-induced antifungal activity of TiO_2_/ZnO nanocomposites on *Candida albicans* biofilms and compared it with that of single TiO_2_ nanoparticles and single ZnO nanowires. It showed that the cell viability in the nano-TiO_2_ treated group was about 4.3 times greater than that in the TiO_2_/ZnO nanocomposites group under visible light (5 h). Herein, the TiO_2_/ZnO nanocomposites considerably enhanced the antifungal activity compared with the corresponding single component nanoparticles [74].

Having all these studies in mind, we made a list of the ZnO nanomaterial-mediated anti-bacterial and anti-fungal applications in Table 1.

### 4.2. ZnO Nanomaterials for Antitumor Applications

In recent years, the incidence of tumors has sharply increased, and the development of anti-tumor drugs is urgent. However, traditional cancer drugs not only kill tumor cells, but also have obvious cytotoxicity to normal cells, and thus the long-term use of large amounts of drugs can easily induce tolerance of the body. Therefore, it is urgent to develop new drugs. Recently, nanodrugs have attracted the favor of researchers because of their superiority in tumor treatment such as large surface area, high drug loading capacity and specific targeting to tumor cells. On the one hand, the nanomaterials can act as novel drug delivery systems. On the other hand, some nanomaterials that have inherent anti-tumor activity can be directly used as the drug. Thus, it is a golden choice for tumor diagnosis and therapy [83]. ZnO nanoparticles are such a promising anti-cancer agent due to their good compatibility and high selectivity [84]. Many studies have shown that ZnO nanoparticles have significant therapeutic effects on HepG2, HeLa, MCF-7, Caco-2 and other tumor cells [85].

#### 4.2.1. Anti-Cancer Mechanisms

**Figure 4:**
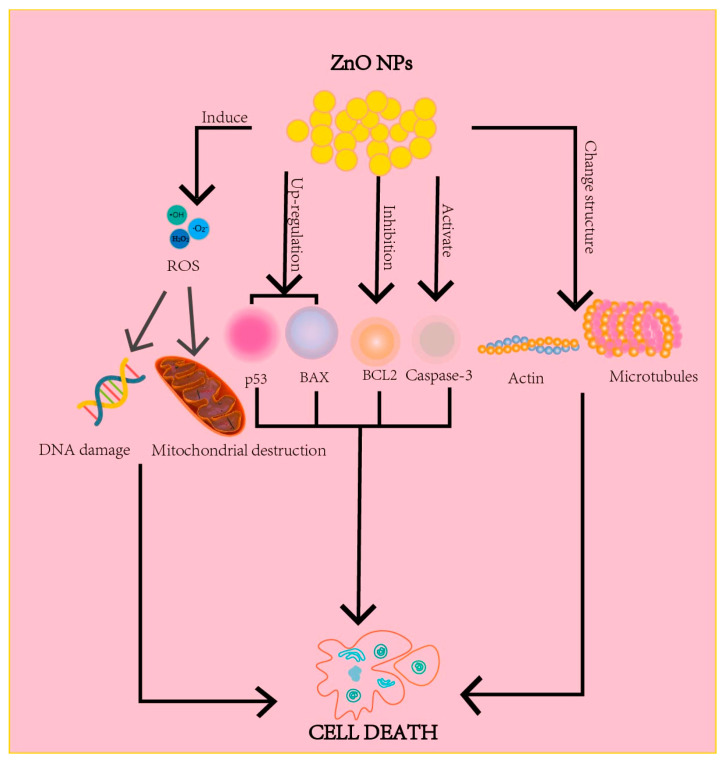
The anti-cancer mechanisms of ZnO nanomaterials.

The main mechanisms of ZnO nanomaterials as anti-cancer agents are: (1) Regulating the gene and protein expressions in tumor cells by the internalization of ZnO nanoparticles so as to induce tumor cell apoptosis [86,87,88]. (2) Generating high levels of ROS to induce cell destruction [86,88]. (3) Altering the structure of actin and microtubules to lead to cell necrosis and apoptosis [86] (Figure 4).

#### 4.2.2. Anti-Cancer Applications

Here, we take the most popular tumor type in nano-ZnO anti-cancer studies, including liver, breast and lung cancer as paradigms, to reveal the promising potential of ZnO nanoparticles for anti-cancer applications.

##### Liver Cancer Treatment

As a highly prevalent malignant tumor, liver cancer threatens the life and health of people around the world. According to the statistics, the number of liver cancer cases in China in 2020 is as high as 410,000, which accounts for fifth place in the incidence of malignant tumors. Additionally, the number of deaths is 390,000, accounting for second place in cancer mortality. ZnO nanomaterials have been investigated in the treatment of liver cancer. Due to the lymphatic drainage obstruction and the insufficient vessel walls, ZnO nanomaterials are more likely to penetrate and remain in the cancerous tissue for a while [87]. Moreover, under physiological conditions, ZnO nanoparticles have a positive surface charge, while cancer cells have a negative membrane potential, and thus the electrostatic interactions drive the interaction of ZnO nanoparticles with cancer cells [88]. Yang et al. studied the effects of ZnO nanomaterials on Huh7 liver cancer cells in vitro. It found that ZnO nanomaterials could promote autophagy, upregulate the caspase3 and p53 expressions and trigger apoptosis, thus inhibiting the growth and proliferation of liver cancer cells [89]. Akhtar et al. found that ZnO nanoparticles promoted ROS production; upregulated the p53, bax and caspase3; downregulated the apoptosis-inhibiting bcl-2 gene; and induced DNA fragmentation, thus eventually causing HepG2 tumor cell apoptosis [90]. Rahimi Kalateh Shah Mohammad et al. investigated the effect of ZnO nanoparticles on Huh-7 and HepG2 cells via green synthesis of ZnO nanoparticles. The results showed that the IC_50_ values of ZnO nanoparticles for HepG2 cells and Huh-7 cells were 40 μg/mL at 72 h and 15 μg/mL at 48 h, respectively, which attributed to the enhancement of P53 and Bax gene expression profiles [91]. To further enhance the therapeutic efficacy of ZnO nanomaterials, researchers often integrate them with other nanoparticles that have similar properties. Alabyadh et al. synthesized ZnO/CeO_2_ nanocomposites via combustion method using metal-organic framework as precursor and explored the toxic effects on HepG2 liver cancer cells [92]. The results showed that the tumor growth inhibition increased with the increase in concentration of ZnO/CeO_2_ nanocomposites. More importantly, the anticancer effect of ZnO/CeO_2_ nanocomposites was better than that of ZnO or CeO_2_ alone.

##### Breast Cancer Treatment

The incidence of breast cancer in China ranks first place in female malignant tumors. The number of breast cancer cases in China increased from 315,000 in 2017 to 336,000 in 2021. As mentioned above, the long-term use of commonly therapeutic drugs such as docetaxel, paclitaxel, adriamycin and epi-amycin will induce tolerance in the body, and the increased dosage leads to more pronounced adverse effects. Therefore, the development of new drugs is necessary. Recently, ZnO nanomaterials presented promising potential for breast cancer treatment. Jasim Makkawi et al. prepared ZnO/CdS composites and investigated their cytotoxicity to breast cancer cells. They found that ZnO/CdS nanocomposites exhibited stronger anticancer activity against mammary gland (MCF-7) cells compared with ZnO nanoparticles alone [93]. This is attributed to the reduced bandgap energy after compositing ZnO nanoparticles with narrow bandgap semiconductor CdS, which increased the production of ROS. Abarna et al. synthesized ZnO/chitosan composites using a simple solid-state method [94]. They found that the number of MCF-7 breast cancer cells exposed to the composite was drastically reduced with an IC_50_ of 34.6% compared with a ZnO alone of 60%, which may be due to the synergistic anti-cancer effect of ZnO nanomaterials and chitosan. Sanad et al. produced structurally stable ZnO-5-FU nanocomposites using physical adsorption for breast cancer cell inhibition [95]. Both the 5-FU and ZnO nanomaterials have anticancer activity. Additionally, the increased ROS in cancer cells facilitate the high permeability, electrostatic interaction and selective cytotoxicity of ZnO. As a result, the incorporation of ZnO into the 5-FU surface significantly increased the killing rate of MCF-7 cells. Bisht et al. used trisodium citrate as a linker to conjugate ZnO nanoparticles and Fe_3_O_4_ to form ZnO-Fe_3_O_4_ magnetic composite nanomaterials (MCPs) [96]. The cytotoxicity of MCPs was investigated on a human breast cancer cell line (MDA-MB-231) and normal mouse fibroblasts (NIH-3T3). The MCPs can utilize both the selectivity of ZnO nanoparticles and the magnetic properties of Fe_3_O_4_ nanoparticles, and thus they had significant selectivity for MDA-MB-231 cells. Moreover, the Fe_3_O_4_ nanoparticles could convert the H_2_O_2_ produced by the interaction of ZnO nanoparticles with cancer cells into ROS, which enhanced the oxidative stress of cells. As a result, it showed that compared with naked ZnO nanoparticles, MCPs exhibited higher selectivity for cancer cells and lower toxicity for normal cells. Vimala et al. synthesized a folic-acid (FA)-modified PEG-ZnO nanosheet (FA-PEG-ZnO NS) and used it as a carrier to loaded anti-cancer drug adriamycin (DOX), which combined the targeted delivery of DOX and photothermal therapy for breast cancer treatment [8]. The DOX-FA-ZnO NS exhibited pH responsive, heat stimulative effects and sustained drug release properties. The experimental results confirmed that combined therapy based on DOX-FA-ZnO NS+NIR possesses a maximum death rate of MDA-MB-231 breast cancer cells compared with that of single chemotherapy or photothermal therapy.

##### Lung Cancer Treatment

Lung cancer is a malignant tumor originating from the bronchial mucosa or glands of the lungs. Its incidence and mortality are increasing extremely fast. WHO data show that China had 816,000 lung cancer cases and about 715,000 deaths in 2020. Lung cancer is currently divided into two categories: small cell lung cancer and non-small cell lung cancer. Additionally, non-small cell lung cancer accounts for approximately 80% of all lung cancers. In recent years, lung cancer therapy based on nano-drugs has developed rapidly, and many relative literature studies can be found. Compared with traditional drug therapy, nanocarrier delivery systems have obvious advantages [97], such as increasing the solubility of the drugs, avoiding drug degradation and reducing the interaction of drugs with non-target tissues so as to reduce the adverse reactions. In this subsection, we will introduce the application of ZnO nanomaterials in lung cancer therapy.

Cisplatin (Cp) and gemcitabine (Gem) are common broad-spectrum hydrophobic antitumor agents in clinic. They have significant efficacy but strong side effects. Based on this, Hu et al. used ZnO nanoparticles as carriers to load Cp and Gem to form ZnONPs(Cp/Gem) [98]. This nanosystem had properties as follows: (1). ZnONPs(Cp/Gem) could upregulate the expression of pro-apoptotic proteins caspase 3, caspase 9 and RARP, and thus promote the apoptosis of cancer cells. (2). ZnONPs(Cp/Gem) increase intracellular ROS, causing oxidative stress in cancer cells. (3). ZnONPs(Cp/Gem) decrease intracellular glutathione, accumulate harmful substances in cells and thus increase cytotoxicity. (4). ZnONPs(Cp/Gem) can damage the mitochondria of cancer cells and enhance cytotoxicity. As a result, the effect of ZnONPs(Cp/Gem) on human non-small cell lung cancer cells A549 was obviously higher than that of Cp, Gem and ZnO nanoparticles alone. Rupa et al. loaded indole-3-carbinol (I3C) on the ZnO nanoparticles under ultrasound to form DM-ZnO-I3C nanoemulsion (DM-ZnO-I3C-NE) [99]. DM-ZnO-I3C-NE can improve the solubility of anticancer drugs in the delivery system so as to improve the drugs’ efficacy. According to the result, DM-ZnO-I3C-NE presented more toxicity in non-small cell lung cancer cells than the free DM-ZnO nanoparticles or I3C; this may be due to the synergistic effect of I3C and Zn^2+^ ion. Zhao et al. synthesized ZnO@polymer nanoparticles, which can be self-assembled into capsule shells to load the hydrophobic drug isotretinoin (ISO), and obtained ZnO-ISO composite [100]. The ZnO@polymer delivery system had a tight structure and was less prone to drug leakage, and it had high pH sensitivity that could decompose under acidic conditions to facilitate drug release. From the results, the antitumor effect of ZnO-ISO composite is better than that of the existing Nintedanib and Crizotinib on the market within a certain drug concentration range. Cai et al. reported a PH-sensitive drug delivery platform based on NH_2_-ZnO quantum dots (QDs) [101]. This nanocarrier system was doped with some substances that have specific binding ability to lung cancer cells. First, the NH_2_-ZnO QDs surface was modified by PEG to improve its stability under physiological fluid. CD44 is a common marker of tumors, and hyaluronic acid (HA) can specifically bind to CD44. Thus, ZnO-PEG was combined with HA molecules to form HA-ZnO-PE.G., Subsequently, DOX drug was loaded onto the HA-ZnO-PE.G., After uptake of the QDs by cancer cells, the pH-sensitive ZnO QDs were dissolved to Zn^2+^ in acidic endosome/lysosome, which triggered the metal–drug complex dissociation and DOX controlled release, thus realizing a synergistic antitumor effect to A549 lung cancer cells by both Zn^2+^ and DOX.

Taken together, we list the ZnO nanomaterial-mediated anti-tumor applications in Table 2.

### 4.3. ZnO Nanomaterials for Anti-Inflammation

Inflammation is a defense response of living tissues to stimulation by various harmful external factors, which is dynamic in injury, anti-injury and recovery. In general, mild inflammation has positive effects on the body, such as limiting lesions and preventing the spread of pathogenic microorganisms to the whole body. Additionally, the fluid and leukocyte exudate can dilute toxins, remove necrotic tissues and repair and restore organ functions. However, severe inflammation has the potential to harm the organism. For example, inflammation can cause denaturation or necrosis of organs, inflammatory exudates can lead to fluid accumulation, mutual adhesions between organs, lesion swelling and compression of the surrounding tissues and organs. Long-term inflammation can cause asthma, rheumatoid arthritis, pancreatitis and other diseases [102]. Very severe acute inflammation may even be directly fatal. Therefore, anti-inflammatory applications are especially important in some sudden disease treatments. The usually used anti-inflammatory drugs are non-steroidal anti-inflammatory drugs (NSAIDs), including aspirin, ibuprofen, diclofenac, indomethacin and so on. However, when NSAIDs inhibit COX-2, they will inevitably restrain COX-1, where the COX-1 is beneficial to the gastrointestinal mucosa. As a result, this causes harmful gastrointestinal reactions, such as epigastric discomfort, nausea, vomiting and even gastric ulcers and bleeding in patients. These obvious adverse effects greatly limit the drug’s application. Moreover, in acute inflammation, the anti-inflammatory, antipyretic and analgesic efficacy of NSAIDs are delayed due to their slow absorption [102]. Therefore, the development of new drugs is particularly meaningful. Recently, many research studies indicated that ZnO nanomaterials exhibit potential in the treatment of inflammatory diseases. We thus discuss this part as follows.

#### 4.3.1. Anti-Inflammatory Mechanisms

**Figure 5:**
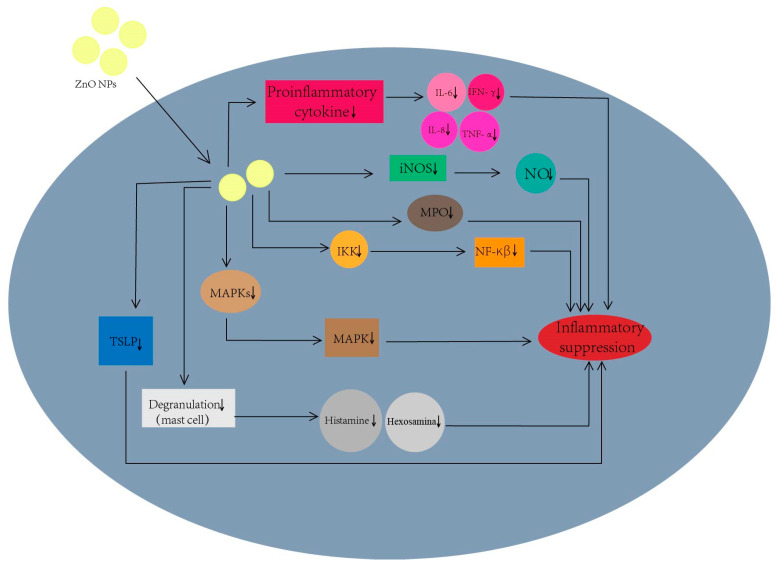
The anti-inflammatory mechanisms of ZnO nanomaterials.

As seen in Figure 5, the main mechanisms of anti-inflammatory effects of ZnO nanoparticles include: (1). Inhibition of inducible nitric oxide synthase (iNOS) protein expression [102]. Nitric oxide (NO) synthesis in the body is related to iNOS, which can participate in the rolling and migration of white blood cells, and can promote the genes’ transcription of pro-inflammatory factors. A high concentration of NO can cause the death of host immune cells. Thereby, the inhibition of iNOS can reduce NO synthesis so as to relieve inflammation in the body. (2). Inhibition of pro-inflammatory cytokine release [102,103,104]. Some cytokines, such as interleukin-6 (IL-6), interleukin-8 (IL-8), interleukin-17 (IL-17), interferon-γ (IFN-γ) and tumor necrosis factor-α (TNF-α), can regulate inflammation. Zn^2+^ can inhibit these cytokine releases to reduce inflammation. (3). Inhibition of myeloperoxidase (MPO) activity [102]. MPO is a heme-containing cationic enzyme, which can act as an inflammation mediator to alter neutrophil function and promote cytokine production. ZnO nanoparticles can effectively inhibit MPO activity and thus exert good anti-inflammatory effects. (4). Inhibition of mitogen-activated protein kinases (MAPKs) [102,103]. The MAPK family involved in the MAPK pathway is closely related to activating and regulating the inflammatory response. ZnO nanoparticles can inhibit the pathway to reduce inflammation. (5). Inhibition of the nuclear factor-κB (NF-κβ) pathway [102,103,105]. NF-κβ can mediate inflammation by promoting the production of pro-inflammatory cytokines and chemokines [102]. ZnO nanoparticles have exactly the IκB kinase (IKK) inhibiting effect to inhibit the NF-κβ pathway and thus exhibit anti-inflammatory activity. (6). Inhibition of mast cell degranulation [102]. In allergic inflammatory diseases such as allergic rhinitis, atopic dermatitis and asthma, mast cells are activated to release a series of inflammatory mediators (e.g., histamine and b-hexosaminidase). ZnO NPs can inhibit these inflammatory mediators to alleviate inflammation. (7). Inhibition of thymic stromal lymphopoietin (TSLP) generation [102,103]. TSLP activates mast cells and stimulates the release of Th2 cytokines to mediate the immune response. ZnO nanoparticles can inhibit the TSLP production to reduce inflammation.

#### 4.3.2. Anti-Inflammatory Applications

The anti-inflammatory properties of ZnO nanoparticles are widely used in biomedical applications. For example, Mahadeva et al. prepared ZnO nanoparticles through a combustion method using *lantana camara* flowers as the fuel, and investigated their anti-inflammatory effect [106]. They showed that ZnO nanoparticles could obviously inhibit Phospholipase A2 (MIC value: 41 μg/mL) to reduce inflammation. This might be attributed to the binding of ZnO nanoparticles and Zn^2+^, which changes the enzyme conformation. Rajakumar et al. explored the anti-inflammatory activity of ZnO nanoparticles via evaluating the protein denaturation inhibition effect [107]. The ZnO nanoparticles presented significant anti-inflammatory potential with an inhibitory IC_50_ of 66.78 μg/mL, which was close to the reference (diclofenac, 62.55 μg/mL). Moreover, ZnO nanomaterials synthesized using *kalanchoe pinnata* [108], *polygala tenuifolia* root extract [109], *Heritiera fomes* and *Sonneratia apetala* extracts [110], *pelargonium odoratissimum* aqueous leaf extract [111], *Trianthema portulacastrum* Linn. [112] and *acacia caesia* bark extract [113] also have obvious anti-inflammatory activity. Xia et al. added ZnO nanoparticles to the diets of weaned piglets, which improved the intestinal flora to inhibit inflammation and thus reduced the incidence of diarrhea in piglets [104]. In ileum, the expression levels of TNF-α, IFN-γ, IL-1β and NF-κB were decreased. In addition, ZnO nanocomposites are also commonly used in anti-inflammatory applications. As a typical example, Yao et al. produced TNTs/ZnO composites via the electrodeposition of ZnO nanoparticles into TiO_2_ nanotubes (TNT) and investigated the anti-inflammatory effect of TNTs/ZnO based on a macrophages model [114]. It was found that TNTs/ZnO can effectively inhibit the macrophage proliferation and adhesion, and thus has the potential to control the inflammatory reaction and prevent chronic inflammation. In addition, ZnO nanocomposites can be indirectly used in anti-inflammatory therapy via acting as catalysts for the synthesis of anti-inflammatory drugs.

Overall, we sum up the ZnO nanomaterial-mediated anti-inflammatory applications in Table 3.

### 4.4. ZnO Nanomaterials for Skin Care

With the development of technology and the progress of industry, climate change is evident and the environmental pollution is serious, so skin problems are becoming more and more common among the general public. The need for skin care and makeup products in daily life has also become particularly urgent. Recently, nanomaterials have been widely used in the skin care industry, such as sunscreens, shampoos, creams, eye creams and color cosmetics [115,116,117,118]. Typically, ZnO nanomaterials have been used in various commercial skin care products, especially sunscreen products, because of their high stability, low irritancy, broad-spectrum absorption and other properties [119].

#### 4.4.1. Sunscreen Mechanism

**Figure 6:**
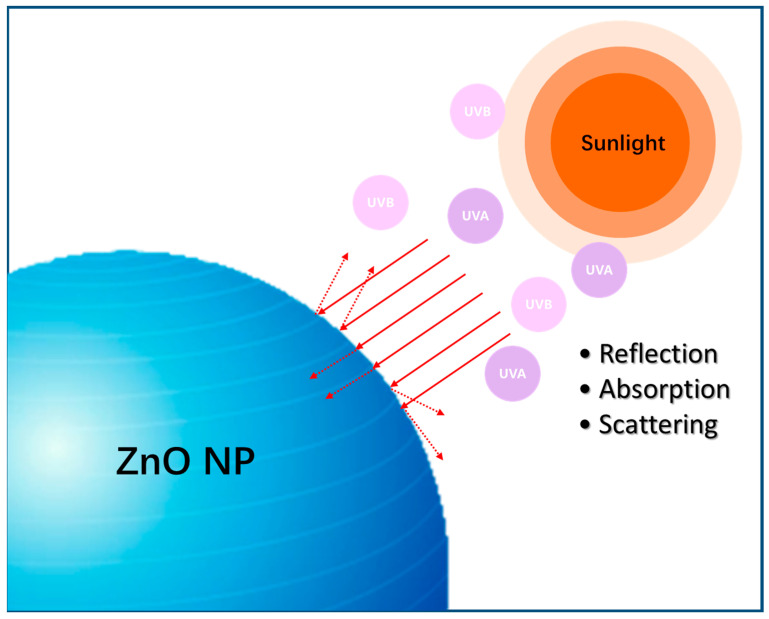
The sunscreen mechanism of ZnO nanomaterials.

The usually so-called sunscreen mechanism is the protection of skin from damage caused by UV-rays in sunlight. As a kind of electromagnetic wave, UV radiation has a non-negligible damage to human skin. Additionally, it can be divided into four sections: (1). UV-A long-wave (320–400 nm); UV-A can penetrate the dermis, lead to melanin increase, damage lipids and collagen, cause skin aging and even induce skin cancer. (2). UV-B medium wave (280–320 nm); UV-B can cause skin sunburn, peeling and erythema, etc. (3). UV-C short wave (200–280 nm); (4) UV-D ultrashort wave (10-200 nm). Generally, UV-C does not invade human skin. Thus, the usual so-called UV injury is UV-A and UV-B injury. Appropriately, ZnO nanoparticles have the potential for absorption, reflection and scattering of UV light in sunlight [119,120]. For example, on the one hand, due to the band gap of ZnO nanoparticles, the electron can be excited and leap in the outer layer orbit by absorbing UV light to achieve the purpose of sun protection. On the other hand, nano-sized ZnO has the ability of UV light scattering and reflecting, and thus exhibits a physical shielding effect that decreases the touch of UV light to skin tissue (Figure 6).

#### 4.4.2. Skin Care Applications

Just under the above-mentioned background, sunscreen products appear more and more frequently in daily life. Research has shown that the regular use of sunscreen can prevent actinic keratosis and squamous cell carcinoma [121]. At present, ZnO inorganic particles have become commonly used physical sunscreens in clinics, and their particle size has gone from traditional micrometer scale to nano-scale in order to improve their transparency and viscosity [122]. Meanwhile, the particle size of ZnO nanoparticles is one of the key factors that affects the sunscreen’s absorption function, where with the decreasing particle size of nanoparticles, the UV absorption performance may be improved [121]. There are many kinds of ZnO-containing sunscreens on the market and the content of ZnO nanoparticles in common commercial sunscreens is about 20% [123]. For example, sunscreens for pregnant women from the well-known Australian brand Kangaroo Mother added ZnO as a physical sunscreen ingredient, which has a theoretical sun protection time of 7.5 h. As another example, the sunscreen products of the well-known Australian brand Invisible Zinc also use ZnO as a main ingredient, which not only has a sun protection function but also has repairing and brightening abilities. Moreover, the sunscreen from American brand Aveeno Kids also contains ZnO, which can reflect UV-A and UV-B in sunlight without skin irritation. In addition, the sunscreen in French brand CeraVe’s moisturizing mineral also contains ZnO to form a protective barrier on the surface of the skin.

Although the sunscreen properties of nanoparticles are favored, it has recently been found that the hazards associated with their penetration into the body cannot be ignored, so we highlight safety studies here to inspire researchers to further optimize the properties of ZnO to improve its safety [124,125,126]. Studies have shown that the main factors affecting the permeation of Zn^2+^ are: (1). pH—Holmes et al. studied the effect of ZnO and Zn^2+^ permeation on the skin, and the results showed that the labile zinc within the viable epidermis increased with the decrease in pH, and the main zinc species are derived from the dissolution of ZnO that exist as Zn^2+^ [127]. (2). Skin barrier integrity—Studies have shown that an intact skin barrier can effectively block the penetration of Zn^2+^. When the skin stratum corneum is damaged, Zn^2+^ in the epidermis is significantly increased. For instance, after 24 h of material used on damaged skin, it is observed that the Zn^2+^ in vivo is up to 60–65 times higher than that used on normal skin. (3). The material retention time on the skin surface—Studies have shown that after 48 h staying on skin, the content of Zn^2+^ in the body is higher than that after 24 h staying. (4). Material size—the smaller the size of the nanoparticles, the greater the ratio of surface area to volume and the faster the dissolution of ZnO. Excessive material penetration will cause high levels of particles in vivo and thereby exhibit cytotoxicity. These factors provide important warnings for the right use of sunscreen containing ZnO nanoparticles. Special attention should also be paid to the fact that sprays cannot contain nanomaterials in order to avoid direct exposure of the lungs to ZnO nanoparticles [128]. Moreover, long-term exposure of ZnO nanoparticles to UV light can induce photocatalytic activity to produce toxic ROS and lose UV-blocking functions [128,129]. In order to synthesize nanoparticles with lower toxicity, Girigoswami et al. coated chitosan (CTS) and polyethylene glycol (PEG) on the surface of ZnO nanoparticles to produce ZnO-CTS and ZnO-PEG, respectively, and the experimental results showed that surface modified-ZnO nanoparticles have lower photocatalytic activity and higher UV-blocking efficiency than bare ZnO nanoparticles [129]. In addition to the preparation of the above two surface modifiers, the researchers also coated aluminum hydroxide, silica and vitamins on ZnO nanoparticles to reduce the toxicity of the materials [129,130].

In addition to sunscreens, ZnO nanomaterials are also used in other skin care products. The beauty industry giants currently using nano-products are mainly L’Oreal, Johnson & Johnson, Avon and Estee Lauder [131]. ZnO nanoparticles are one kind of nano-product used by these companies. The significant antibacterial and anti-inflammatory properties make ZnO nanoparticles used in moisturizers. The ability for cell proliferation and collagen formation make them of use in face and eye creams. Additionally, the appropriate concentration of zinc can facilitate skin wound healing and protects the body from UV-A and UV-B damage, which thereby are applied in body lotions. Additionally, the function of improving product stability makes ZnO nanoparticles doped in eyeliner [131]. Furthermore, different morphologies of ZnO nanomaterials have different applications. ZnO nanocrystals with a diameter less than 100 nm are applied in toothpaste to protect tooth enamel. ZnO nanocapsules can load active substances such as lipid mixtures and lipophilic substances to penetrate into the deep dermis, and thus are often used as emulsions or hydrogels in personal care products. ZnO nanoemulsions with uniformity and low viscosity are widely used in anti-aging products [123,130,131,132].

### 4.5. ZnO Nanomaterials for Bioimaging

Nowadays, semiconductor nanomaterials are increasingly used in the field of bioimaging, but the commonly used cadmium quantum dots (QDs) are limited due to their significant cytotoxicity [133]. In contrast, ZnO QDs have low toxicity and Zn is a trace element in the human body, which has a promising future in bioimaging [88,134,135,136,137,138]. Nowadays, many types of ZnO nanoparticles have been developed, such as ZnO QDs and ZnO nanoflowers [133,139,140,141]. The photoluminescence spectrum of ZnO includes UV emission and visible light emission, where the broad visible emission of ZnO nanomaterial makes it more suitable for bioimaging [140].

#### 4.5.1. Imaging Mechanisms

**Figure 7:**
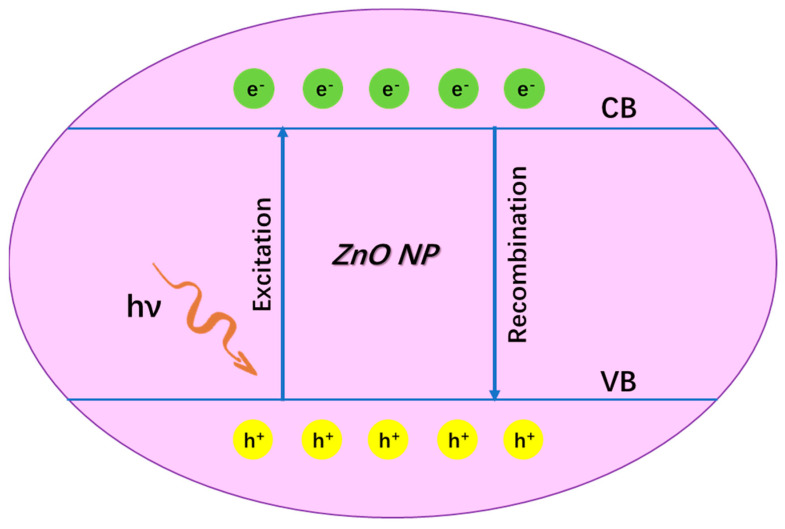
The bio-imaging mechanisms of ZnO nanomaterials.

The luminescent properties of ZnO nanomaterials are important for their use in imaging. The possible mechanisms of UV and visible emission of ZnO are described briefly below: (1). The recombination of photo-generated electrons with holes in the VB or in traps near the VB. (2). The recombination of electrons in shallow trap with a hole in a deep trap and the recombination of electrons in the singly ionized oxygen vacancies with the photo-generated holes in the VB [140,142]. As a result, the visible emission of ZnO QD is essentially due to the electrons leaping between energy levels [140] (Figure 7).

#### 4.5.2. Imaging Applications

The surface defects, the band gap width and the physical properties of ZnO nanomaterials can affect their imaging applications. Herein, many researchers have modified the surface of ZnO nanoparticles, widened the band gap and developed new synthesis methods to improve their utilization value, and the ZnO nanoparticles can even be used as carriers to load colorant for assistant imaging [139,140,141,142,143,144,145,146,147,148,149]. Zang et al. synthesized ZnO nanoflowers using a biphasic reaction method. It had a band-edge absorption feature at λ = 360 nm (3.44 eV) that was greater than the bulk (~3.36 eV), which might be attributed to quantum-confinement [141]. In order to improve the water solubility of the materials that are good for bio-labeling, they exchanged the surface ligand with aminoethanol ethyl ester hydrochloride (AET), which can convert the hydrophobic surface of nanoparticles into a hydrophilic surface, but still can retain its photoluminescence performance. The biological cell labeling results showed that most Hela cells were successfully labeled by AET–ZnO nanoparticles and the corresponding blue emissions could be imaged under fluorescent microscopy. Wu et al. synthesized ZnO QDs using a soft chemical method and capped them with 3-aminopropyl trimethoxysilane (Am) to protect the core nanocrystals [150]. Subsequently, the ZnO QDs were capped with TiO_2_ via sol-gel method to synthesize ZnO-TiO_2_ QDs, where the nanoparticles prepared by the sol-gel method exhibit strong surface defects and thus can induce emission at longer wavelengths. They used mung bean seedlings as experimental objects and monitored the fluorescence emission intensity of seedling cross-sections to evaluate the bioimaging performance of ZnO nanomaterials. The results showed that the emission intensity of seedlings treated with TiO_2_-ZnO QDs was 18 times more than that of the untreated seedlings. Furthermore, it found that TiO_2_-ZnO QDs had a higher quantum yield than the standard fluorescein material. Senthilkumar et al. prepared a ZnO-ZnS core–shell structure where the nanostructure possessed a thin ZnS layer around ZnO nanoparticles [133]. The formation of the core–shell structure confined the charge carriers within the core band gap, which resulted in a significant increase in the photoluminescence intensity and thus was beneficial to imaging. Manaia et al. synthesized Mg-ZnO QD using a hydrolysis-condensation reaction and compared the photoluminescence effect between pure ZnO and Mg-ZnO QD [140]. They found that the luminescence performance of ZnO QD doped with Mg^2+^ was significantly improved. With the increase in Mg^2+^ proportion, both the emission and absorption spectra presented a blue shift phenomenon that increased the luminescence. When the doping concentration of Mg^2+^ in ZnO was 20 mol%, the luminescence exhibited a maximum with the quantum yield of 64%, which is significantly higher than that of pure ZnO suspension (10%).

With all of this research in mind, we list the ZnO nanomaterial-mediated bio-imaging applications in Table 4.

### 4.6. ZnO Nanomaterials for Food Packaging

With the frequent use of various food additives, antioxidants and antimicrobial agents in food, food-borne diseases also appear frequently, and people are paying more and more attention to food quality and food safety. Therefore, the development of new types of food additives with good biosafety is necessary. Zn^2+^ is a trace element required by the human body, and ZnO acting as a zinc supplement has been widely used in the food industry [151]. The FDA has classified ZnO as a safe material (GRAS) [152]. To avoid zinc deficiency, ZnO is often incorporated into food products as a food additive. In addition, the significant antimicrobial activity, high stability, low toxicity and large surface area of ZnO nanoparticles make them able to be used as active packaging [153,154,155,156,157,158,159]. This section will mainly describe the application of ZnO nanoparticles in active food packaging.

#### 4.6.1. Material Performance Evaluation

Active packaging is food packaging that can give food a longer shelf-life and bring better economic efficiency to businesses. As a type of active packaging, antimicrobial packaging can extend the shelf-life of food by inhibiting and slowing the growth of microorganisms in the food [153]. Unlike traditional food preservatives that are directly added into food, antimicrobial packaging employs the antimicrobial agents to load onto the packaging material and gradually diffuse into the food matrix to provide antimicrobial effects that improve food safety [153]. The performance of packaging materials is usually evaluated in the following indicators [152,153,160]: (1). Mechanical properties—this includes the tensile strength (TS) and elongation at break (EAB) of the film. Generally speaking, the higher the TS and EAB, the stronger anti-damage ability and the better elasticity of the material. (2). Water solubility and swelling—this is the indicator of water resistance of bio-composite film. (3). Barrier properties—this includes water vapor permeability (WVP) and oxygen permeability (OP). WVP is the study of water transfer on the film; lower WVP values means better barrier performance of the packaging material and lower food moisture loss. Additionally, the smaller the OP, the better the oxygen separation performance of the packaging material. (4). Transparency—the lower the transparency, the better blocking performance of the material to UV-visible light. (5). Antibacterial activity. (6). Anti-oxidation properties.

#### 4.6.2. Food Packaging Application

Chitosan (CS) is a biopolymer with significant antimicrobial activity, biocompatibility and low toxicity, which is now widely used as a direct coating for meat and fruit products [160]. Al-Naamani et al. once made CS-ZnO nanocomposites coat onto polyethylene (PE) film and investigated their antimicrobial efficacy compared with that of CS-coated PE [160]. The experimental results showed that compared with the control group, the counts of *Escherichia coli*, *Salmonella* and *Staphylococcus aureus* decreased only 10-fold after incubation with the CS-coated PE, while the CS-ZnO PE could completely inactivate as well as prevent the growth of food pathogens. Liu et al. used CS, ZnO nanoparticles and antioxidant bamboo leaves (AOB) to make active films and investigated their bioactivity and physical properties [161]. It was found that the adding of AOB enhanced the light-blocking effect of CS-ZnO film, particularly UV light blocking. Additionally, the light-blocking property improved with the increase in AOB concentration, which was probably due to the significant absorption of UV by the flavonoid compounds in AOB. The radical scavenging effects of material on 2,2-diphenyl-1-bitter hydrazide (DPPH) and 2,2-azido-bis-(3-ethylbenzothiazolidine-6-sulfonic acid) (ABTS) were also increased, which was mainly due to the antioxidant capacity of the flavonoid compounds in AOB. Meanwhile, the synergistic effect of AOB with ZnO greatly enhanced the antimicrobial effect against *E. coli* and *S. aureus*. Yadav et al. produced CS-ZnO@gal complex membranes using CS, ZnO nanoparticles and gallic acid (gal). It was found that the performance of CS-ZnO@gal membranes was greatly improved compared with that of pure CS membranes [152]. Firstly, the addition of ZnO@gal into CS improved the TS and EAB, which may be due to the fact that ZnO nanoparticles can fill the space of the CS matrix well and establish a strong interfacial interaction with the CS matrix. Secondly, the solubility and swelling of the CS-ZnO@gal membrane were reduced, which can be attributed to the denser and better sealing of the film. In addition, the antibacterial and antioxidant properties of the membranes were enhanced due to the doping of ZnO nanoparticles and gal, and the higher content of ZnO@gal, the higher the antioxidant properties of the membranes. Moreover, the WVP and OP values of CS-ZnO@gal membranes were lower compared with those of pure CS membranes, which were attributed to the ZnO@gal doping that made the network structure of the membrane tighter. Considering the environmental impact of packaging materials, Sarojini et al. used sesame oil-based polyurethane (PU) and CS as raw materials and incorporated different concentrations of ZnO nanoparticles to prepare biodegradable food packaging films [162]. The mechanical properties, water resistance and antimicrobial activity of the PC3-5Z film (containing 5% ZnO nanoparticles, a 3:1 weight ratio of PU and CS) were higher than those in pure CS film, which might be attributed to the denser structure of the film with the addition of PU and ZnO and the significant antimicrobial activity of ZnO. Additionally, the biodegradability of PC3-5Z was significantly enhanced, which possessed an 80% degradation rate within 28 days compared with plain PU films. This biodegradable food packaging film not only meets the expected requirements of packaging films, but is also very friendly to the environment. In addition, ZnO nanoparticles can also be combined with other natural-, organic- or biopolymers, such as acetylated cellulose nanocrystals [163], polylactic acid [157], starch-poly vinyl alcohol [164], mangosteen peel extract [165] and polycarbonate [166]. They can effectively enhance the mechanical properties, barrier properties, antibacterial and antioxidant properties, biodegradation properties and even pH sensing properties of the materials, which are promising escorts for food safety.

In all, we now list the ZnO nanomaterial-mediated food packaging applications in Table 5.

## 5. Biosafety of ZnO Nanomaterials

The assessment of the biosafety (or toxicity) of nanomaterials is a prerequisite for their practical application. Recently, diverse toxicity research results of ZnO nanoparticles have been reported in several species such as mammalian cells, animals, plant microbes and algae. It should be pointed out that we only discuss the toxicity of ZnO nanoparticles to mammalian cells and animals, but not to plants or other species, here because this paper primarily focuses on the biological applications of ZnO under human body exposure, which is more closely related to human health rather than eco-environmental influence. For instance, Sarkar et al. evaluated the bio-safety of mycosynthesized ZnO-NPs (75 ± 5 nm) using cytotoxicity and genotoxicity assays in human lymphocyte cells. The analysis of membrane integrity and mitochondrial dehydrogenase activity indicated that the cytotoxicity was significant at treatment doses of 500 µg/mL and above. Comet assay revealed that the obvious genotoxicity occurred at the highest concentration of 1000 µg/mL [167]. Of course, biosafety evaluation only at the cellular level is not enough; the in vivo data are more persuasive. Thus, researchers conduct more animal experiments in later studies. For example, Yu et al. investigated the oral acute toxicity of ZnO nanoparticles in rats. They showed that no obvious acute toxicity was observed after oral administration of ZnO nanoparticles (86.3 ± 23.8 nm, 100 mg/kg) for 14 days. Nevertheless, transcriptomic responses in the livers were affected, which showed that the ZnO nanoparticles up-regulated the metabolic process and metal biding [168]. Jung et al. studied the effect of the interaction between ZnO nanoparticles and food matrices on oral toxicity. The results indicated that after oral administration of ZnO nanoparticles (78 ± 26 nm, 100 mg/kg) interacting with glucose (5%) or albumin (5%) for 14 consecutive days, no acute toxicity was found in rats via the histopathological, hematological and serum biochemical analyses, revealing their low oral toxicity [169]. Kong et al. assessed the oral acute toxicity and oral long-term toxicity of unmodified ZnO nanoparticles (50 nm) in mice, respectively [170,171,172]. For the oral acute toxicity study, the experiments of clinical signs, body weights, organ weight, serum biochemistry, hematology, gross pathology, histopathology and mortality were measured, which demonstrated that the toxicity of 50 nm ZnO nanoparticles was greater than bulk ZnO, but similar to 20 nm ZnO nanoparticles, and the LD50 was 5177 mg/kg·bw [170]. For the oral long-term toxic effects study, it indicated that after a 90-day exposure, the unmodified 50 nm ZnO nanoparticles caused hepatic and renal function impairment, anemia and antioxidant system imbalance of mice. Remarkable toxic effects were observed under the doses > 80 mg/kg. Additionally, the lowest observed adverse effect level was 40 mg/kg·bw [171]. In addition, the unmodified 50 nm ZnO nanoparticles can cause lipid metabolism disorder, reproductive toxicity and hyperlipidemia to male mice through oxidative injury in long-term exposure [172].

As can be seen from the above case, bio-toxicity is influenced by many factors including dose, size, exposure time and so on. Thus, it can be predicted that more caution needs to be taken for ZnO nanoparticles, particularly with their use in food additives and skin care products that directly expose the human body, which need to strictly control the use parameters.

## 6. Conclusions and Outlook

The multifunctionality of ZnO nanomaterials provides a good opportunity for biological application. In this review, we systematically introduce the recent advances of ZnO nanomaterial-mediated biological applications and corresponding action mechanisms, which cover anti-bacterial, anti-tumor, anti-inflammatory, bioimaging, skin care and food packaging uses. Meanwhile, we also give a brief summary of the structure-dependent physicochemical properties, the common synthesis methods and the biosafety of ZnO nanoparticles. Note that although the fact that significant progress and achievements have been obtained in this field, challenges still exist that we have to face and tackle, such as the following:

(i). Biosafety evaluation and improvement need further and deeper research. Although toxicity studies of ZnO nanoparticles have been extensively published, the reports about long-term effects are still not enough. It is not difficult to see that the acute toxicity of ZnO nanoparticles is low according to many reports. Thus, the chronic toxicity and long-term effects are more valuable to be concerned with. Meanwhile, the genetic toxicity evaluation of ZnO nanoparticles also needs to be further strengthened, which is closely related to the health of future generations. In addition, for the improvement of material biosafety, we can make material modifications such as alterations of the surface reactive property to improve its biocompatibility and minimize the adverse effects [173].
(ii). Strengthening toxicity mechanisms study and establishing standardization. To date, the understanding of the toxicity mechanisms of ZnO nanoparticles is not deep enough, and the research results are diverse, even contradictory, in different models. The absence of key nanotoxicity information and the lack of consensus among researchers on the experimental evaluation index is unbeneficial to regulation control and enforcement in use. Therefore, the experimental protocols of toxicity mechanisms are demanded, and guidance and standardization for nanotoxicity evaluation that includes cytotoxicity, in vivo toxicity, genetic toxicity and so on should be established.
(iii). The optimization of the synthesis. On the one hand, for existing mature synthesis methods, one can optimize the experimental parameters such as pH, temperature and ingredient proportion to rationally regulate the physicochemical properties of ZnO nanoparticles so as to obtain the particle with desired size, shape and specific surface area, etc. On the other hand, the yield demand of ZnO nanoparticles has increased dramatically due to their large-scale commercial use. Thus, simple, low-cost and eco-friendly new synthesis methods such as popular green synthesis are currently an urgent task.
(iv). Proper surface functionalization of ZnO nanoparticles. The surface of a nanoparticle is closely related with its dispersibility, biocompatibility and biotoxicity in a physiological environment. Meanwhile, the modification of the surface with various functional biomolecules, such as aptamers and antibodies, is helpful to improve the selectivity and targeting ability to pathogenic tissue and so on. Thus, the proper surface functionalization of ZnO nanoparticles is significant to enhance its therapeutic efficacy for diseases such as tumors.
(v). Deepening the research on the action mechanism. It is not difficult to see that the action mechanisms are more than one path of each ZnO nanomaterial-mediated application. For example, the ZnO-mediated antibacterial mechanisms cover not only ROS generation but also Zn^2+^ release, disturbance of the cell membrane and so on. So, which path is dominant? Or is there an inner link between these paths? Questions such as this need to be further explained.

## Figures and Tables

**Table 1:** A list of representative nano-ZnO-mediated anti-bacterial and anti-fungal applications.

Nanomaterials	Synthetic Method	Particle Size	Type of Microbe	Anti-Microbial Outcomes	Ref.
GPTMS-ZnO nanoparticle	Sol-gel	Varying sizes from 5.3 to 38.2 nm	*Staphylococcus aureus*	Antibacterial activity increased with decreased particle size	[3]
ZnO nanocrystalline	HEBM	Varying sizes from 20 to 250 nm	*Staphylococcus*, *Streptococcus*, *Micrococcus*, *Escherichia Coli*, *Enterobacter* and *Pseudomonas*	Antibacterial activity increased with decreased particle size	[62]
ZnO nanoparticle	Bio-mediated solution combustion	11–26 nm of crystallite size	*Pseudomonas aeruginosa* and *Staphylococcus aureus*	ZnO nano-flower exhibited excellent photocatalytic activity over the ZnO with other morphology	[59]
ZnO nanoparticle	Bio-mediated method using *Aloe vera* and *Hibiscus sabdariffa* plant extracts	9–18 nm	*Escherichia coli*, *Klebsiella pneumoniae*, *Pseudomonas aeruginosa* and *Staphylococcus aureus*	More efficient bactericidal activity was found in nanoparticles via biosynthesis than those from chemical synthesis	[68]
Ag-decorated ZnO nanoparticle	Chemical reduction	30–50 nm	*Staphylococcus aureus* and *Escherichia coli*	The hybrid of nanoparticles improved the antibacterial performance	[69]
ZnO/graphene oxide composites	Covalent bonding	170 nm of ZnO	*Escherichia coli*, *Salmonella typhimurium*, *Bacillus subtilis* and *Enterococcus faecalis*	The composite action of materials improved antibacterial properties	[2]
Chitosan-stabilized ZnO nanoparticles	Microwave heating	50–70 nm	*Staphylococcus aureus* and *Escherichia coli*	Chitosan-stabilized ZnO nanoparticles exhibited excellent antibacterial activity	[70]
ZnO nanoparticles	Sol-gel	Less than 50 nm	*Candida albicans*	Effective antifungal effect was observed	[75]
ZnO nanoparticles	Homogeneous precipitation method	~30 nm	*Candida albicans*	The antifungal activity was positively correlated with the ZnO concentration	[80]
ZnO nanoparticles	Bio-mediated method using *Citrus hystrix* leaf extract	26−69 ± 0.5 nm	*Candida albicans* and *Aspergillus niger*	Exhibition of good antifungal activity at a higher concentration of 5 μg/mL	[76]
ZnO nanoparticles	Bio-mediated method using *Azadirachta indica* leaf extract	29.024 nm	*Aspergillus niger*	Maximum suppression of lychee fruit rot was obtained at the dose of 1.0 mg/mL	[72]
HDPE/ZnO-MMT nanocomposites	A combination of simple ion exchange and reduction reactions	~20 nm	*Aspergillus niger*	Significant antifungal properties were found at 5 wt% of loading	[79]
5%Pd-doped nano-ZnO	Thermal decomposition method	35 nm	*Aspergilus niger* and *Candida albicans*	Pd-doped nano-ZnO increased the antifungal activity over pure nano-ZnO	[73]
TiO_2_/ZnO nanocomposites	Chemical vapor deposition	50−100 nm	*Candida albicans* biofilms	TiO_2_/ZnO nanocomposites enhanced antifungal activity more than single component	[74]

**Table 2:** A list of the representative nano-ZnO-mediated anti-tumor applications.

Nanomaterials	Synthetic Method	Particle Size	Type of Cancer Cells	Anti-Cancer Outcomes	Ref.
ZnO nanoparticles	/	14.13 ± 0.92 nm	Huh7 liver cancer cells	Effectively inhibited liver cancer cells’ growth and proliferation	[89]
ZnO nanoparticles	Co-precipitation	21.59 ± 4.89 nm	HepG2 liver cancer cells	The HepG2 cell viability was significantly decreased to 39% at dose of 15 μg/mL	[90]
ZnO nanoparticles	Bio-mediated method using *H. officinalis* plant extracts	10−100 nm	HepG2 and Huh7 liver cancer cells	IC_50_ values for HepG2 and Huh-7 cells were 40 μg/mL at 72 h and 15 μg/mL at 48 h, respectively	[91]
ZnO/CeO_2_ nanocomposites	Combustion	31.9 nm	HepG2 liver cancer cells	The anticancer effect of ZnO/CeO_2_ nanocomposites was better than that of single component	[92]
ZnO/CdS nanocomposites	Chemical synthesis	/	MCF-7 breast cancer cells	The anticancer effect of ZnO/CdS nanocomposites was better than that of ZnO alone	[93]
ZnO/chitosan nanocomposite	Solid-state synthesis	25−31.67 nm	MCF-7 breast cancer cells	ZnO/chitosan nanocomposites exhibited excellent anti-tumor activity compared with ZnO alone	[94]
ZnO-Fe_3_O_4_ magnetic nanocomposite	Ex situ conjugation	44.05 ± 1.2 nm	MDA-MB-231 breast cancer cells	ZnO-Fe3O4 showed preferential toxicity to breast cancer cells but low ctoxicity towards noncancerous cells	[96]
DOX-FA-ZnO NS	EDC/NHS coupling and physical absorption	160 nm	MDA-MB-231 breast cancer cells	The combined therapy based on DOX-FA-ZnO NS possessed maximum death rate of breast cancer cells compared with single therapy	[8]
ZnONPs(Cp/Gem)	Mixing	~20 nm	A549 lung cancer cells	The effect of ZnONPs(Cp/Gem) on lung cancer cells was higher than that of single component	[98]
DM-ZnO-I3C nanoemulsion	Ultrasonication	239.6 ± 6.13 nm	A549 lung cancer cells	DM-ZnO-I3C-NE presented more toxicity in lung cancer cells than free DM-ZnO or I3C	[99]
ZnO−ISO nanocomposite	Self-assembly	~100 nm	A549 lung cancer cells	The antitumor effect of ZnO-ISO composite was better than the existing drugs of Nintedanib and Crizotinib on the market	[100]
HA-ZnO-DOX	EDC/NHS coupling	~3 nm of ZnO	A549 lung cancer cells	HA-ZnO-DOX exhibited a good synergistic antitumor effect to lung cancer cells	[101]

**Table 3:** A list of ZnO nanomaterial-mediated anti-inflammatory applications.

Nanomaterials	Synthetic Method	Particle Size	Inflammatory Indicators	Anti-Inflammatory Outcomes	Ref.
ZnO nanoparticles	Combustion method	21.4–27.2 nm	Phospholipase A2	Presenting high inhibition for Phospholipase A2	[106]
ZnO nanoparticles	Bio-mediated method using *Andrographis paniculata* leaf extract	The sizes of 96–115 and 57 ± 0.3 nm with spherical and hexagonal shapes, respectively	Inhibiting protein denaturation	Presenting significant inhibitory effects with IC_50_ of 66.78 μg/mL	[107]
ZnO nanoparticles	/	23.0 nm	IFN-γ, IL-1β, TNF-α and NF-κB	Inhibiting the expression of the inflammatory factor	[104]
ZnO nanoparticles	Bio-mediated method using *kalanchoe pinnata* leaf extract	24 nm	TNF-α, IL-1β, IL-6 and COX-2	Blocking the production and release of inflammatory mediators	[108]
ZnO nanoparticles	Bio-mediated method using *Polygala tenuifolia* root leaf extract	33.03−73.48 nm	iNOS, COX-2, IL-1β, IL-6 and TNF-α.	Suppressing both mRNA and protein expressions of iNOS, COX-2, IL-1β, IL-6 and TNF-α.	[109]
ZnO nanoparticles	Bio-mediated method using *Heritiera fomes* and *Sonneratia apetala* extracts	40–50 nm	Inhibiting protein denaturation	Presenting significant inhibitory effects with IC_50_ of 63.25 μg/mL	[110]
ZnO nanoparticles	Bio-mediated method using *pelargonium odoratissimum* aqueous leaf extract	21.6 nm	Human red blood cells’ membrane stabilization	Promoting the stabilization of the red blood cells’ membrane	[111]
ZnO nanoparticles	Bio-mediated method using *Trianthema portulacastrum* Linn.	10–20 nm	Human red blood cells’ membrane stabilization, protein denaturation and proteinase inhibitor activity	Good membrane stabilization efficiency against human red blood cell membrane, effectively preventing albumin denaturation and obvious proteinase inhibitory activity	[112]
ZnO nanoparticles	Bio-mediated method using *acacia caesia* bark extract	32.32 nm	COX	Inhibiting the expression of the COX	[113]
TNTs/ZnO	Electrodeposition	Inner diameter 50 nm	Macrophage	TNTs/ZnO can effectively inhibit the macrophage proliferation and adhesion	[114]

**Table 4:** A list of representative nano-ZnO-mediated bio-imaging applications.

Nanomaterials	Synthetic Method	Particle Size	Labeling Model	Bio-Imaging Outcomes	Ref.
AET–ZnO nanoparticles	Double-phase reaction	30 nm	Hela cells	Hela cells were successfully labeled and imaged via blue emission	[141]
ZnO-TiO_2_ QDs	Sol-gel method	~50 nm	Mung bean seedling plant cells	Good bio-imaging capability on plant cells	[150]
ZnO@Polymer core–shell nanoparticles	Sol-gel method	3–4 nm	Human hepatoma cells (QGY 7763)	The ZnO QDs penetrated into the living cells and exhibited bright fluorescence imaging	[143]
CoFe_2_O_4_-ZnO core–shell nanoparticles	Wet chemical synthesis	11.6 ± 1.8 nm	MCF-7 (human breast cancer cells)	The cells containing the core–shell nanoparticles were visible in blue, green and red emission	[144]
ZnO/HPEI nanocomposites	Chemical synthesis	3 nm of ZnO	COS-7 cells	The water-soluble ZnO/HPEI nanocomposites could easily be endocytosed by the COS-7 cells without transfection reagent and exhibited excellent biological imaging behavior	[145]
ZnO/SiO_2_ core–shell nanoparticles	Wet chemical synthesis	60 nm of ZnO core and 7–10 nm of the width shell	NIH 3 t3 fibroblast cells	This nanoparticle showed high visible florescence even at lower concentrations	[146]
ZnO@alizarin nanoparticles	Solvothermal synthesis	47.4 nm of ZnO	Amyloid oligomers and plaques	Effective imaging properties	[148]
Graphene/FA-ZnO nanocomposite	Co-precipitation and conjugation method	22.4–35.4 nm of diameter and 84.1–154 nm of length.	Swiss albino mice implanted with Ehrlich Tumor	The position of fluorescence in the tumor confirmed that the nanocomposite was prepared for in vivo tumor targeting.	[149]

**Table 5:** A list of representative nano-ZnO-mediated food packaging applications.

Nanomaterials	Synthetic Method	Particle Size	Evaluation Indicators	Outcomes	Ref.
CS-ZnO PE	Stirring	~55 nm of ZnO	Antimicrobial efficacy	Completely inactivated and prevented the food pathogens’ growth	[160]
CS-ZnO	Stirring	50 ± 10 nm of ZnO	Light-blocking effect, radical scavenging effects, antimicrobial effect	UV light blocking was the most obvious, DPPH and ABTS radical scavenging effects was obvious and antimicrobial effect against *E. coli* and *S. aureus* was powerful	[161]
CS-ZnO@gal complex	Solution casting	19.2 nm of ZnO@gal	TS, EAB, solubility, swelling, antibacterial and antioxidant effect, WVP and OP	Improved the TS and EAB, reduced the solubility and swelling, enhanced the antibacterial and antioxidant effect and decreased WVP and OP	[152]
PC3-5Z film	Solvent casting	~55 nm of ZnO	Mechanical properties, water resistance, antimicrobial activity, biodegradability	All these properties of PC3-5Z film were better than those of plain PU films	[162]
PLA/ACNC/ZnO films	Solution casting	50 nm of ZnO	UV blocking, mechanical strength, oxygen and water vapor barrier, antibacterial activity	Improved the UV barrier with a slight decrease in transparency, mechanical and barrier properties. The tensile strength, oxygen barrier and water vapor barrier increased compared with those of pure PLA film	[163]
PLA/ZnO	Melt mixing	100−500 nm of ZnO	Mechanical and barrier properties, antimicrobial property	Good mechanical properties, a slight increase in water vapor and excellent antimicrobial property against *E. Coli*	[157]
PSNZJ	Solvent casting	/	Water barrier, UV barrier, mechanical and antimicrobial properties	This film enhanced the water barrier, UV barrier, mechanical and antimicrobial properties.	[164]
SPI/MPE/ZnO composite film	Solution casting	30 ± 10 nm of ZnO	Mechanical strength, water vapor transmission, UV-blocking, antioxidant and antibacterial properties	The composite film exhibited excellent UV-blocking, antioxidant and antibacterial properties against *Escherichia coli* and *Staphylococcus aureus*	[165]
ZnO/polycarbonate nanocomposite	Blade coating	15–20 nm of ZnO	Antibacterial properties, UV-blocking properties and hydrophobicity	Good UV-blocking capability, enhanced bacteriostatic action against *S. aureus* and *E. coli* and enhanced hydrophobic character.	[166]

## Data Availability

Not applicable.
